# Cell Factory Design and Culture Process Optimization for Dehydroshikimate Biosynthesis in *Escherichia coli*

**DOI:** 10.3389/fbioe.2019.00241

**Published:** 2019-10-09

**Authors:** Si-Sun Choi, Seung-Yeul Seo, Sun-Ok Park, Han-Na Lee, Ji-soo Song, Ji-yeon Kim, Ji-Hoon Park, Sangyong Kim, Sang Joung Lee, Gie-Taek Chun, Eung-Soo Kim

**Affiliations:** ^1^Department of Biological Engineering, Inha University, Incheon, South Korea; ^2^STR Biotech Co., Ltd., Chuncheon-si, South Korea; ^3^Department of Molecular Bio-Science, Kangwon National University, Chuncheon-si, South Korea; ^4^Green Chemistry and Materials Group, Korea Institute of Industrial Technology, Cheonan-si, South Korea; ^5^Green Process and System Engineering Major, Korea University of Science and Technology (UST), Daejeon, South Korea

**Keywords:** dehydroshikimate, cell factory design, *Escherichia coli*, culture process, production optimization

## Abstract

3-Dehydroshikimate (DHS) is a useful starting metabolite for the biosynthesis of muconic acid (MA) and shikimic acid (SA), which are precursors of various valuable polymers and drugs. Although DHS biosynthesis has been previously reported in several bacteria, the engineered strains were far from satisfactory, due to their low DHS titers. Here, we created an engineered *Escherichia coli* cell factory to produce a high titer of DHS as well as an efficient system for the conversion DHS into MA. First, the genes showing negative effects on DHS accumulation in *E. coli*, such as *tyrR* (tyrosine dependent transcriptional regulator), *ptsG* (glucose specific sugar: phosphoenolpyruvate phosphotransferase), and *pykA* (pyruvate kinase 2), were disrupted. In addition, the genes involved in DHS biosynthesis, such as *aroB* (DHQ synthase), *aroD* (DHQ dehydratase), *ppsA* (phosphoenolpyruvate synthase), *galP* (D-galactose transporter), *aroG* (DAHP synthase), and *aroF* (DAHP synthase), were overexpressed to increase the glucose uptake and flux of intermediates. The redesigned DHS-overproducing *E. coli* strain grown in an optimized medium produced ~117 g/L DHS in 7-L fed-batch fermentation, which is the highest level of DHS production demonstrated in *E. coli*. To accomplish the DHS-to-MA conversion, which is originally absent in *E. coli*, a codon-optimized heterologous gene cassette containing *asbF, aroY*, and *catA* was expressed as a single operon under a strong promoter in a DHS-overproducing *E. coli* strain. This redesigned *E. coli* grown in an optimized medium produced about 64.5 g/L MA in 7-L fed-batch fermentation, suggesting that the rational cell factory design of DHS and MA biosynthesis could be a feasible way to complement petrochemical-based chemical processes.

## Introduction

Aromatic amino acid biosynthetic pathways have long been a potential source for commercially relevant chemicals with diverse industrial applications (Tzin and Galili, 2010). One of the core metabolites that facilitate the production of useful intermediates in aromatic amino acid biosynthetic pathways is 3-dehydroshikimate (DHS), a key intermediate in the biosynthesis of shikimic acid (SA), muconic acid (MA), vanillin, and protocatechuate (PCA) (Li et al., 2019). This group of chemicals has drawn much attention for the major feedstock for food, drugs and commodity chemicals (Chen and Nielsen, 2013; Becker and Wittmann, 2015). Bio-based production of these compounds is a promising alternative to the current petrochemical-based production routes that are a cause of environment and energy concerns (Jan and Cavallaro, 2015). MA is easily converted into adipic acid and terephthalic acid, which find universal application in the synthesized nylons and polymer polyethylene terephthalate (PET) industries (Draths and Frost, 1994; Kruyer and Peralta-Yahya, 2017). In addition, SA is an initial precursor for the production of a well-known anti-virial compound named oseltamivir phosphate, also known as Tamiflu®.

**Figure 1:**
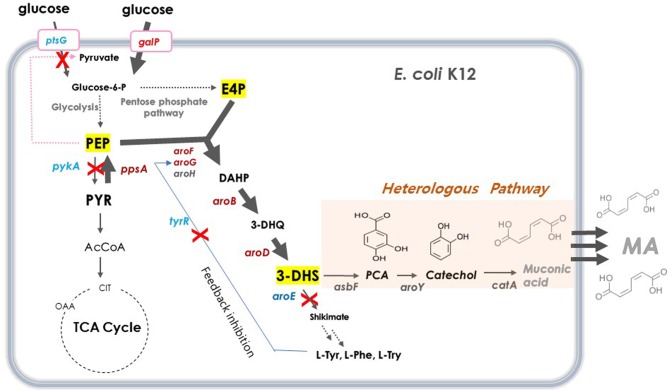
Schematic overview of metabolic pathway for DHS biosynthesis in *E. coli*. Red crosses denote disrupted genes and bold blue arrows denote steps that are overexpressed. Dashed arrows represent two or more steps. G6P, glucose-6-phosphate; PEP, phosphoenolpyruvate; PYR, pyruvate; AcCoA, acetyl-coenzyme A; OAA, oxaloacetate; CIT, citrate; E4P, erythrose4-phosphate; DAHP, 3-deoxy-D-arabinoheptulosonate-7-phosphate; DHQ, 3-de-hydroquinate; DHS, 3-dehydroshikimate; SA, shikimic acid; PCA, protocatechuate; CA, catechol, MA, muconic acid. *ptsG*, glucose specific sugar: phosphoenolpyruvate phosphotransferase; *galP*, D-galactose transporter; *zwf*, glucose-6-phosphate 1-dehydrogenase; *tktA*, transketolase; *tyrR*, tyrosine dependent transcriptional regulator; *pykF*, pyruvate kinase 1; *pykA*, pyruvate kinase 2; *ppsA*, phosphoenolpyruvate synthase; *pckA*, phosphoenolpyruvate carboxykinase; *aroG*, DAHP synthase; *aroF*, DAHP synthase; *aroB*, DHQ synthase; *aroD*, DHQ dehydratase; *aroE*, shikimate dehydrogenase: *asbF*^*Eopt*^, 3-dehydroshikimate(DHS) dehydratase; *pcaG/H*, codon-optimized PCA deoxygenase; *aroY*^*Eopt*^, codon-optimized PCA decarboxylase; *catA*^*Eopt*^, codon-optimized CA 1,2 -dioxygenase.

DHS has a six-member carbon ring with two asymmetric carbons, which is difficult and costly to synthesize chemically (Richman et al., 1996; Nishikura-Imamura et al., 2014). On the other hand, DHS is a natural metabolite found in the shikimate pathway in plants, fungi, and bacteria, making its production feasible through microbial fermentation (Li et al., 1999). DHS biosynthesis in *E. coli* starts from glucose, via the glycolysis pathway, a central carbon metabolism (Figure 1). Two intermediates, phosphoenolpyruvate (PEP) and erythrose-4-phosphate (E4P), are converted to DHS via 3-deoxy-D-arabino-heptulosonate-7-phosphate (DAHP) and dehydroquinate (DHQ) by DAHP synthase (*aroG, aroF*, and *aroH*), DHQ synthase (*aroB*), and DHQ dehydratase (*aroD*).

For several decades, many approaches have been applied to increase the carbon flux toward shikimate biosynthetic pathway for the production of several aromatic compounds. For example, there was an attempt to solve the feedback inhibition issue of DAHP synthase to improve the production of aromatic compounds (Pittard and Yang, 2008; Wang et al., 2013). Another strategy involved the inactivation of the PEP-consuming PTS system and use of another PEP-independent glucose uptake pathway, as PEP is an important precursor of an aromatic compound (Patnaik et al., 1995; Chandran et al., 2003; Johansson et al., 2005). Yet another approach was to eliminate the pyruvate kinases involved in PEP conversion to pyruvate, thereby enhancing the pathway that converts pyruvate to PEP (Patnaik et al., 1995; Chandran et al., 2003; Johansson et al., 2005; Meza et al., 2012; Rodriguez et al., 2013). Combinations of these strategies are typically used to improve the productivity of aromatic compounds (Yi et al., 2002; Wang et al., 2013; Rodriguez et al., 2014; Martínez et al., 2015; Lee et al., 2018).

Recently, we engineered a *Corynebacterum glutamicum* strain to overproduce MA from glucose, through redesign of the aromatic amino acid biosynthetic pathway (Lee et al., 2018). Through heterologous expression of a codon-optimized PCA decarboxylase gene cluster under a strong promoter, and deletions of several key genes involved in MA intermediate bypass routes, an optimized pathway for DHS-PCA-CA-MA was successfully constructed in *C. glutamicum*. Optimization of culture media and processes using the MA-producing *C. glutamicum* strain were performed to achieve a significantly increased titer of MA (Lee et al., 2018).

In this study, we generated engineered *E. coli* strains to overproduce both DHS and MA, through manipulation of several key genes involved in DHS biosynthesis and heterologous expression of codon-optimized foreign genes involved in DHS-to-MA conversion. These redesigned DHS-overproducing and MA-overproducing *E. coli* strains, grown in optimized media, produced the highest levels of both DHS and MA production in 7L fed-batch fermentations, indicating that the rational microbial cell factory design of DHS and MA biosynthesis could be an alternative way to complement petrochemical-based chemical processes.

## Materials and Methods

### Bacterial Strains, Media, and Culture Conditions

The bacterial strains and plasmids used in this study are listed in Table 1. *E. coli* DH5a was used as the cloning host, and *E. coli* K12 & AB2834 were used as parent strains. *E. coli* strains for genetic manipulations were grown in Luria-Bertani (LB) medium at 37 or 30°C. For DHS production in small-scale culture, a single colony was inoculated in LB medium at 30°C for 15 h, and secondary culture was inoculated with 1% (v/v) in the same medium at 30°C for 6 h. The miniature culture was grown using 1.3 mL of *E. coli* production medium (EPM) in a 24-well cell culture plate, at 220 rpm under 30°C, for 4 days. The composition of the EPM for DHS and MA production was: glucose (5 g/L), glycerol (10 g/L), yeast extract (2.5 g/L), tryptone (2.5 g/L), KH_2_PO_4_ (7.5 g/L), MgSO_4_ (0.5 g/L), (NH_4_)_2_SO_4_ (3.5 g/L), NH_4_Cl (2.7 g/L), Na_2_SO_4_ (0.7 g/L), Na_2_HPO_4_•12H_2_O (9 g/L), and a trace metal solution (1 mL/L). The trace metal solution contained FeSO_4_•7H_2_O (10 g/L), CaCl_2_•2H_2_O (2 g/L), ZnSO_4_•7H_2_O (2.2 g/L), MnSO_4_•4H_2_O (0.5 g/L), CuSO_4_•5H_2_O (1 g/L), (NH_4_)_6_Mo_7_O_24_•4H_2_O (0.1 g/L), and Na_2_B_4_O_7_•10H_2_O (0.02 g/L).

**Table 1:** Plasmids and strains used in the study.

**Strains**	**Relevant characteristics**	**References**
*E. coli* AB2834	K12 *ΔaroE*	YALE univ.
*E. coli* Inha24	*E. coli ΔtyrR*	This study
*E. coli* Inha29	*E. coli* Inha24 *ΔptsG*	This study
*E. coli* Inha52	*E. coli* Inha29 *ΔpykA*	This study
*E. coli* Inha95	*E. coli* Inha52 *ΔlacI:*: P*lac_aroB_aroD_*P*lac_ppsA_galP*	This study
*E. coli* Inha99	*E. coli* Inha52 *ΔlacI:*: P*lac_aroB_ aroD*	This study
*E. coli* Inha103	*E. coli* Inha52 *ΔlacI:*: P*lac_aroB_aroD_*P*lac*_*aroG*_*aroF*P*lac_ppsA_galP*	This study
*E. coli* InhaM101	*E. coli* Inha103/pMESK1	This study
*E. coli* InhaM104	*E. coli* Inha103/pMESK4	This study
*E. coli* InhaM105	*E. coli* Inha103/pMESK5	This study
*E. coli* InhaM106	*E. coli* Inha103/pMESK6	This study
*E. coli* InhaM107	*E. coli* Inha103/pMESK7	This study
*E. coli* InhaM108	*E. coli* 4 Inha103/pMESK8	This study
**Plasmids**		
T-vector	T&A cloning vector	RBC Real biotech.
pUC18	Standard backborn vector for MA expression	Clontech
pET21b(+)	Protein expression vector for *E. coli*	Novagen
pKOV	The suicide vector containing the *Bacillus subtilis sacB* gene and temperature sensitive pSC101 replication origin	Addgene
pKOV_*tyrR*	pKOV containing a PCR fragment for the markerless disruption of the *tyrR* gene	This study
pKOV_*ptsG*	pKOV containing a PCR fragment for the markerless disruption of the *ptsG* gene	This study
pKOV_*pykA*	pKOV containing a PCR fragment for the markerless disruption of the *pykA* gene	This study
pKOV_*lacI*	pKOV containing a PCR fragment for the markerless disruption of the *lacI* gene	This study
pKOV_BD	pKOV_*lacI* derivative for P*lac_aroB_aroD* integration into *lacI* region	This study
pKOV_PABD	pKOV_*lacI* derivative for P*lac*_*ppsA_galP*_P*lac*_*aroB_aroD* integration into *lacI* region	This study
pKOV_PAGFBD	pKOV_*lacI* derivative for P*lac*_*ppsA_galP*_P*lac*_*aroG_aroF*_P*lac*_*aroB_aroD* integration into *lacI* region	This study
pMESK1	pUC18 modification vector including Lac promoter *asbF^*Eopt*^- aroY^*Eopt*^- catA^*Eopt*^*	This study
pMESK4	pUC18 modification vector including *oppA* promoter *asbF^*Eopt*^- aroY^*Eopt*^- catA^*Eopt*^*	This study
pMESK5	pUC18 modification vector including *dps* promoter *asbF^*Eopt*^- aroY^*Eopt*^- catA^*Eopt*^*	This study
pMESK6	pUC18 modification vector including *rmf* promoter *asbF^*Eopt*^- aroY^*Eopt*^- catA^*Eopt*^*	This study
pMESK7	pUC18 modification vector including *fusA* promoter *asbF^*Eopt*^- aroY^*Eopt*^- catA^*Eopt*^*	This study
pMESK8	pUC18 modification vector including *rnpB* promoter *asbF^*Eopt*^- aroY^*Eopt*^- catA^*Eopt*^*	This study

### Construction of Plasmids and Strains

All constructed plasmids for integration and disruption are listed in Table 1. Plasmid DNA extraction and purification were performed using a commercial kit (TIANGEN). For PCR, the Pfu DNA polymerase and HIFI DNA polymerase (TransGen) were used. For disruption and substitution of chromosomal genes, suicide vector pKOV (Addgene, USA) was used, which has the *sacB* gene for providing a markerless system. The constructed plasmids were transformed into *E. coli* AB2834 by electroporation.

For disruption of *tyrR, ptsG, pykA*, and *lacI* genes, homologous DNA fragments were PCR-amplified with primer sets R1-R4, G1-G4, A1-A4, and I1-I4, respectively, which were then inserted into the pKOV vector, generating pKOV-R, pKOV-G, pKOV-A, and pKOV-I, respectively. For overexpression of several genes, T-lac was generated by inserting the lac promoter and RBS into T-vector. The lac promoter and RBS were amplified with primer sets Plac1-Plac2 and RBS1-RBS2 from pUC18 and the pET-21b(+) vector. DNA fragments containing *E. coli aroB, aroD, galP, ppsA, aroG*, and *aroF* genes were amplified with primer sets B1-B2, D1-D2, P1-P2, A3-A4, G3-G4, and F1-F2, respectively, and the amplified DNA fragments were inserted into the T-lac vector, generating T-BD, T-PA, and T-GF. Subsequently, Plac_*aroB_aroD* cassette was amplified with primer sets BD1-BD2 and T-BD as a template, and the amplified cassette was inserted into pKOV-I, generating pKOV-BD. The Plac_*aroB_aroD* and Plac_*galP_ppsA* cassettes were amplified with primer sets BD1-BD3 and PA1-PA2, using T-BD and T-PA as a template, respectively, and the amplified cassettes were inserted into pKOV-I, generating pKOV-PABD. The Plac_*aroG_aroF* cassette was amplified with primer sets GF1-GF2, using T-GF as a template, and the amplified cassette was inserted into pKOV-PABD, generating pKOV-PAGFBD (Supplementary Table 1).

**Figure 2:**
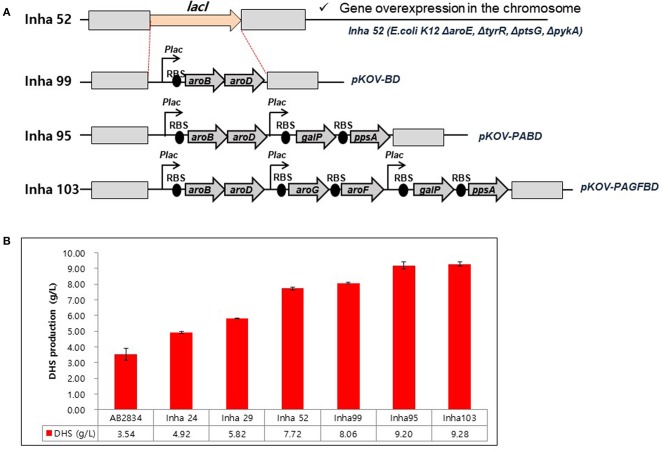
**(A)** Construction of *E. coli* mutants for overproduction of DHS. **(B)** HPLC analysis of DHS production yields in DHS-producing *E. coli* strains. *E. coli* cells were cultured in 1.3 mL of EPM at 30°C for 96 h. Data represent averages and standard deviations from two independent experiments.

A series of *E. coli* DHS-producing strains were constructed using the host strain AB2834, which was constructed by the markerless disruption of the *aroE* gene. The *tyrR* gene in AB2834 was disrupted using plasmid pKOV-R, generating the Inha 24 strain. Subsequently, the *ptsG* and *pykA* genes were disrupted from Inha 24 using plasmids pKOV-G and pKOV-A, generating strains Inha 29 and Inha 52, respectively. Markerless integration of *aroB* and *aroD* genes, under the control of the *lac* promoter, were integrated at *LacI* gene locus into strain Inha 52 using plasmid pKOV-BD, generating the strain Inha 99 (Figure 2A). Using the plasmid pKOV-PABD, the *aroB, aroD, ppsA*, and *galP* genes, under the control of the lac promoter, were integrated into strain Inha 52, generating strain Inha 95. The *aroB, aroD, ppsA, galP, aroG*, and *aroF* genes, under the control of the lac promoter, were chromosome-integrated using plasmid pKOV-PAGFBD in Inha 52 strain, generating strain Inha 103.

### RNA Sequencing

For RNA sequencing, *E. coli* strains AB2834 and Inha52 were cultured in LB media for seeding, and in 500 mL of EPM media for the main production. Growth curves were generated, and each strain was sampled at the early stage of exponential phase (AB2834, 4 h; Inha52, 7 h) as well as the early stage of stationary phase (AB2834, 9 h; Inha52, 12 h). RNeasy mini prep kit (Qiagen, CA) was used for total RNA isolation, according to the manufacturer's instructions. DNaseI (TaKaRa, Japan) was used to eliminate any potential chromosomal DNA contamination. RNA-Seq was performed by ChunLab Inc., Korea.

### Metabolite Analysis

The *E. coli* cells were removed from cultures by centrifugation and culture supernatant was filtered using a membrane filter. The metabolites were separated by HPLC using an Aminex HPX-87H column (Bio-rad, Japan). The mobile phase was 2.5 mM H_2_SO_4_, and a flow rate of 0.6 mL/min was used for metabolites. The column had to be heated at a temperature of 65°C for detection of metabolites. MA and DHS were analyzed at wavelengths of 262 and 236 nm, respectively. Organic acids, including succinic acid, acetic acid, formic acid, and lactic acid, were detected at wavelengths of 210 nm.

### Fed-Batch Fermentation

The first growth culture was carried out in a conical tube containing 5 mL of LBG medium (30°C and 200 rpm). After 12 h of culture, 0.2 mL of the culture broth was inoculated in a 250-mL baffled flask containing 20 mL of culture medium, and cultured at 30°C and 200 rpm in an incubator. After 6 h, the second culture broth was inoculated into a 7-L fermenter (1% v/v inoculum). Production culture was carried out using the PB4-md5 medium in a 7-L fermenter (working volume: 2 L). The PB4-md5 medium includes: 30 g/L glucose, 10 g/L glycerol, 15.75 g/L yeast extract, 21.375 g/L tryptone, 5.25 g/L KH_2_PO_4_, 1 g/L MgSO_4_ · 7H_2_O, 0.8 g/L citric acid, 1 mL/L trace metals, and 200 μg/L thiamine hydrochloride. The feeding medium includes: 600 g/L glucose, 100 g/L yeast extract, 20 g/L MgSO_4_ · 7H_2_O, and 5 mL/L trace metal. Phosphate was not added to the feeding medium, to allow for the regulation of cell growth. The pH levels were 7.0 in each culture (10N NaOH, 3M HCl), and the DO level was maintained above at 30% by controlling the agitation, aeration, and feeding rates. The configuration of the fermenter was as follows: two impellers with turbine and marine impeller, ring-type sparger with 12 holes, top-driven, and 160 mm tank diameter. The feeding medium was supplied when glucose was depleted, using a peristaltic pump.

### Construction of a Gene Cassette Involved in DHS-to-MA Conversion

*E. coli* Inha M101 was prepared by transformation of a plasmid pMESK1 into Inha M103. pMESK1 was prepared by ligation of an *AsbF*^*Eopt*^ *-aroY*^*Eopt*^ *-catA*^*Eopt*^ cassette to pUC18. Codon-optimized gene versions for increased protein expression in *E. coli* were synthesized from Cosmo Genetech, Korea. Digestion of pUC57_M1 with *Xba*I and *Hind*III liberated a 3.3-kb *asbF*^*Eopt*^ *-aroY*^*Eopt*^ *-catA*^*Eopt*^ fragment. Plasmid pUC18 was digested at the same restriction site. Subsequent ligation of *asbF*^*Eopt*^ *-aroY*^*Eopt*^ *-catA*^*Eopt*^ to pUC18 resulted in pMESK1. The ligation of *the asbF*^*Eopt*^ *-aroY*^*Eopt*^ *-catA*^*Eopt*^ cassette was verified by restriction enzyme digestion analysis. To select the promoter for expression of heterologous gene cluster, the intergenic region of *rnpB, fusA, oppA, dps*, and *rmf* genes (upstream region of the start codon) was amplified by PCR and cloned into pMESK1 digested by *Eco*RI and *Xba*I.

## Results

### Redesign of 3-Dehydroshikimate Biosynthetic Pathway in *Escherichia coli*

We used the *E. coli* AB2834 strain as a parental host for DHS accumulation, which is an *aroE* mutant strain lacking shikimate dehydrogenase (Draths and Frost, 1994). To further improve DHS accumulation, we first disrupted the key genes involved in DHS accumulation in *E. coli* AB2834. The *tyrR* repressor, which exerts negative control on the transcription of *aroG* and *aroF* genes with two aromatic amino acids (L-phenylalanine and L-tyrosine), was disrupted (Bongaerts et al., 2001). The *tyrR*-disruption mutant strain (named Inha 24) accumulated 4.92 g/L of DHS in the culture medium after 96 h, whereas the parental strain produced 3.54 g/L of DHS under the same culture condition. Next, the *ptsG* gene, one of the phosphotransferase system genes that consume PEP for glucose uptake, was disrupted in the Inha 24 strain (named Inha 29) to increase the availability of PEP, a DHS precursor. The Inha 29 strain produced 5.82 g/L of DHS under the same culture condition. Finally, the *pykA* gene, encoding a pyruvate kinase, was also disrupted in the Inha 29 strain (named Inha 52), leading to a production of 7.72 g/L of DHS, signifying a more than 2-fold improvement in production yield relative to the parental strain (Figure 2B).

RNA sequencing-based comparative transcriptome analysis between the parental strain and Inha52 revealed that the transcription levels of the DHS biosynthetic pathway genes such as *aroB* (encoding DHQ synthease) and *aroD* (encoding DHQ dehydratase) were significantly reduced in the Inha52 strain (data not shown). To overcome this limitation, both *aroB* and *aroD* genes were overexpressed under the control of the *lac* promoter via an integration plasmid pKOV-BD, generating the strain Inha 99 (Figure 2A). The Inha 99 strain, harboring an extra copy of *aroB* and *aroD* genes, produced 8.06 g/L of DHS (Figure 2B). In addition, Inha 95 was constructed using pKOV-PABD to overexpress the *galP* and *ppsA* genes as well as *aroB* and *aroD* genes in the Inha 52 strain (Figure 2A). The *galP* gene, encoding D-galactose transporter, was overexpressed to improve the level of glucose uptake. The *ppsA* gene, encoding a phosphoenolpyruvate synthase, was also overexpressed to increase the availability of PEP. Inha 95, overexpressing the *aroB, aroD, galP*, and *ppsA* genes, produced 9.20 g/L of DHS (Figure 2B). Subsequently, *aroG* and *aroF* genes, encoding DAHP synthase, were additionally overexpressed in strain Inha 95, generating the Inha 103 strain (Figure 2A). The Inha 103 strain, harboring extra copies of *aroB, aroD, galP, ppsA, aroG*, and *aroF* genes under the control of the *lac* promoter, produced 9.28 g/L of DHS, which is 20% higher DHS yield than that of strain Inha 52, and 2.6-fold higher than that of the parental strain (Figure 2B; Supplementary Table 2). Thus, the DHS production yield of our recombinant strains increased gradually through serial disruptions and overexpression of the genes involved in DHS synthesis.

### Fed-Batch Fermentation for DHS Production

**Figure 3:**
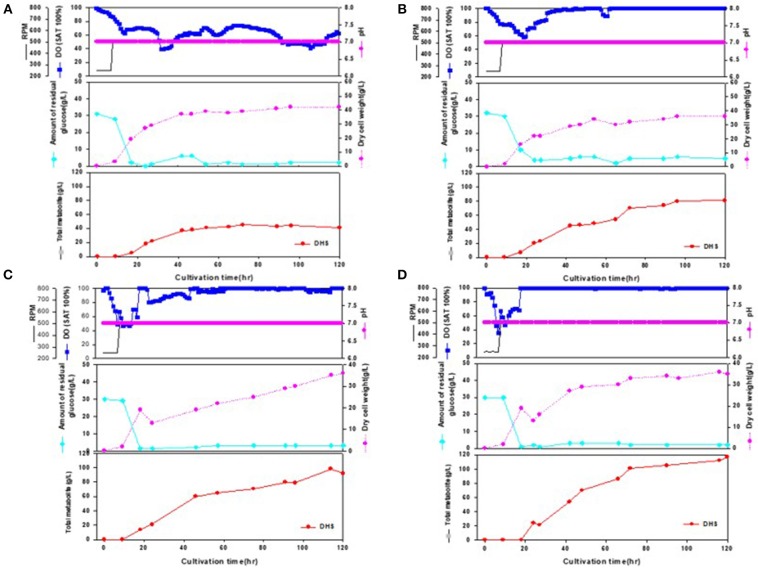
Time-course profiles of cell growth (DCW), glucose, organic acid, and metabolite production by strains in the 7-L fermenter. **(A)** Inha 24: The feeding medium was sequentially injected at culture periods of 17, 19, 24, 27, 42, 47, and 54 h, at a rate of 0.1176, 0.1568, 0.1176, 0.1568, 0.0784, 0.0522, and 0.1176 mL/min, respectively. **(B)** Inha 29: The feeding medium was sequentially injected at culture periods of 17, 19, 24, 42, 47, 54, and 89 h, at a rate of 0.0392, 0.1176, 0.1568, 0.0784, 0.0522, 0.1176, and 0.0784 mL/min, respectively. **(C)** Inha 52: The feeding medium was sequentially injected at culture periods of 17, 19, 42, 47, 54, 65, and 89 h, at a rate of 0.0392, 0.1176, 0.0784, 0.0522, 0.1568, 0.1176, and 0.0522 mL/min, respectively. **(D)** Inha 103: The feeding medium was sequentially injected at culture periods of 18, 24, 42, and 66 h at a rate of 0.1306, 0.1568, 0.1306, and 0.1045 mL/min, respectively.

During the statistical medium optimization process in shake-flask cultures using the metabolically engineered *E. coli* cells, composition of the production medium was found to be a very important factor for enhanced DHS production. Sufficient supply of dissolved oxygen during the entire fermentation period was also observed to be crucial, as revealed by higher DHS production yield according to increase in oxygen mass transfer rate (kLa) through increment of agitation speed (rpm). Therefore, we intended to develop a fed-batch operation process that could efficiently control the producers' fermentation physiology, simultaneously overcoming the oxygen-limited culture conditions caused by high cell density in bioreactor fermentations. The fed-batch culture was carried out using the abovementioned four strains, i.e., Inha 24, Inha 29, Inha 52, and Inha 103. The optimized medium from flask culture was again optimized for the C/N ratio in the fermenter. The DHS production yield was confirmed by fed-batch culture based on this medium. Scaled-up operations from shake flasks to fermenters are largely affected by the operating conditions such as gas-liquid mass transfer, shear stress, mixing time, antifoam, pH regulation, and power input. A phosphate-limited fed-batch culture was performed to regulate cell growth. After the glucose level were almost depleted in the fermentation broth, feeding medium was continuously injected using a peristaltic pump to maintain below 10 g/L to prevent the production of organic acids by residual glucose. As shown in Figure 3, DO levels could be successfully maintained above 30% during the whole fermentation time in the respective fed-batch fermentation, thereby leading to overcoming the undesirable oxygen-limited conditions due to relatively high cell density. It is well-known that when *E. coli* cells become oxygen-limited, cellular metabolism rapidly changes, producing proteases that can degrade foreign proteins, thus lowering the production ability of the transformed *E. coli* cells.

All four strains consumed glucose for 17 h and started feeding from this time onwards. As a result, in the Inha 24 strain, the cells showed steady growth, and the DCW (Dry cell weight) and production yield declined 48 h after the incubation; the DCW was about 42 g/L and the DHS was about 45 g/L. In the same culture condition, the Inha 29 strain exhibited the maximum cell dry weight of about 37 g/L and a DHS level of 74 g/L. In the Inha 52 strain, DCW was lower than that of the Inha 29 strain, but DHS production yield was as high as 81 g/L, implying that there is a competitive relationship between cell growth and DHS production. Although DHS is a primary metabolite, its production in the engineered strains exhibited typical secondary metabolite patterns (Figure 3). In aromatic amino acid processes, *E. coli* and *C. glutamicum* have characteristic metabolites that suppress cell density at certain concentrations (Cheng et al., 2012; Rodriguez et al., 2014; Lee et al., 2018). Finally, in the Inha 103 strain with enhanced flux to DHS, the DCW was about 35 g/L, slightly lower than that of Inha 52, while the production yield of DHS had increased by 44% to 117 g/L. The specific production yield (Yp/x) was 1.07 g DHS/g DCW for the Inha 24 strain, 2 g DHS/g DCW for the Inha 29 strain, 2.25 g DHS/g DCW for the Inha 52 strain, and 3.34 g DHS/g DCW for the Inha 103 strain. The specific production yield of the Inha 103 strain was 3.1-times higher than that of Inha 24 (Table 2; Supplementary Figure 3).

**Table 2:** A, 7-L lab-scale fermentation; B, 50-L pilot-scale fermentation; *P*_*f*_, maximum DHS production (g IA/L); *X*_*f*_, maximum dry cell weight (g DCW/L); *S*_*f*_, final residual glucose concentration (g glucose/L); *Q*_*p*_, average volumetric DHS production rate (g DHS/L/h); *q*_*p*_, average specific DHS production rate (g DHS/g DCW/h); *Y*_*p*/*x*_, specific DHS production (g DHS/g DCW); *Y*_*p*/*s*_, DHS production yield based on glucose (g DHS/g glucose); *Y*_*x*/*s*_, DCW yield based on glucose (g DCW/g glucose).

	***P_***f***_***	***X_***f***_***	***S_***f***_***	***Q_***p***_***	***q_***p***_***	***Y_***p*/*x***_***	***Y_***p*/*s***_***	***Y_***x*/*s***_***
Inha 24	45	42	2	0.375	0.009	1.07	0.21	0.19
Inha 29	74	37	2	0.616	0.017	2.0	0.27	0.14
Inha 52	81	36	5	0.675	0.019	2.25	0.34	0.15
Inha 103	117	35	2	0.975	0.028	3.34	0.39	0.12

### Heterologous Expression of the Genes Involved in DHS-to-MA Conversion

**Figure 4:**
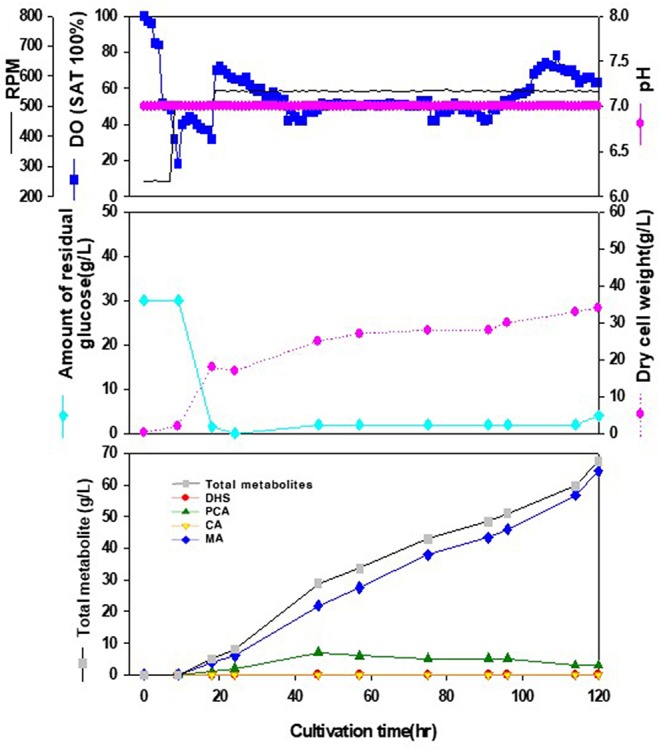
Time-course profiles of cell growth (DCW, pink closed diamond), glucose (cyan closed diamond), organic acid, and metabolite production by the engineered *E. coli* InhaM104 in the 7-L fermenter. The feeding medium was sequentially injected at culture periods of 18, 24, and 46 h, at a rate of 0.0392, 0.0784, and 0.0653 mL/min, respectively. Closed gray square, total metabolite; red closed diamond, DHS (3-dehydroshikimic acid); green closed diamond, PCA (protocatechuate); yellow closed triangle, CA (catechol); blue closed diamond, MA (*cis-cis* muconic acid).

We tried to produce muconic acid, another valuable compound, through heterologous expression of the genes involved in DHS-to-MA conversion in the DHS-overproducing *E. coli* strain. Previously, three foreign genes (*aroZ, aroY*, and *catA*) were reported to be responsible for the DHS-to-MA conversion in *E. coli* (Draths and Frost, 1994, Figure 1). Similarly, we generated a codon-optimized single operon construct containing three genes, *asbF* from *Bacillus thuringiensis* (encoding DHS dehydratase for DHS-to-PCA), *aroY* from *Klebsiella pneumoniae* (encoding PCA decarboxylase for PCA-to-CA), and *catA* from *Acinetobacter calcoaceticus* (encoding catechol 1,2-dioxygenase for CA-to-MA). Genes *asbF*^*Eopt*^*, aroY*^*Eopt*^, and *catA*^*Eopt*^ were cloned into modified pUC18 under the control of the lac promoter, thus generating pMESK1. To optimize the heterologous foreign genes expression in a DHS-overproducing *E. coli* strain, we searched for a naturally-suitable promoter *via* comparative transcriptome analysis between the parental strain and Inha 52 (Supplementary Figure 1). Among the gene expression profiles during the culture duration, five promoters were selected according to their transcription strength, and each selected promoter was cloned to control the foreign gene cassette (Supplementary Figure 2A). The *E. coli* strains expressing each promoter from pMESK4 to pMESK8 were named *E. coli* Inha M104–M108, respectively, and were tested to determine whether MA accumulated in their culture broths. The production level of MA varied, depending on the promoter, as shown in Supplementary Figures 2B and 4. The highest MA titer was observed in strain Inha 103, expressing *oppA* gene promoter, which encodes periplasmic oligopeptides-binding protein in *E. coli*. It yielded up to 1,789 mg/L of MA, an 8-fold increase compared with strain Inha M101 (Plac_ *asbF*^*Eopt*^ *-aroY*^*Eopt*^ *-catA*^*Eopt*^), which showed an MA titer of 223 mg/L. DHS was accumulated in relatively large amounts in two strains (P*lac* and P*dps*), and only a small amount of MA was produced in the other two strains (P*rmf* and P*rmpB*). In the 7-L fermenter, the Inha 103 strain containing the *asbF*^*Eopt*^ *-aroY*^*Eopt*^ *-catA*^*Eopt*^ operon cassette under the control of *oppA* promoter produced 64.5 g/L MA with little accumulation of other intermediates (Figure 4).

## Discussion

In general, strategies to redesign metabolic pathways constitute the primary goal of metabolic engineering, which is a prerequisite to produce certain valuable compounds. This strategy has been used to engineer the shikimate pathway for producing a number of useful aromatic products in *E. coli*. For example, Chandran et al. reported an engineered *E. coli* strain that achieved 84 g/L of shikimate with a yield of 0.33 mol/mol glucose (Chandran et al., 2003). In addition, Wang et al. reported a 48 g/L yield of L-tryptophan, with a conversion ratio of 21.87% from glucose (Wang et al., 2013). It was previously reported that the introduction of heterologous biosynthetic genes such as *aroZ, aroY*, and *catA* into the *aroE*-deleted *E. coli* led to the accumulation of MA up to 36 g/L via benzene-independent pathway (Niu et al., 2002). In addition, various microbial cell factory strategies were also purposed to enhance production of MA or shikimic acid (Table 3) (Noda and Kondo, 2017; Bilal et al., 2018a).

**Table 3:** Recent overview of engineered microbial strains for enhanced shikimate and MA biosynthesis.

**Product**	**Organism/strain**	**Feedstock**	**Culture style**	**Titer**	**Fermentation duration (h)**	**References**
Shikimate	*Escherichia coli*	Glycerol + glucose	Batch	1.78 g/L	36	Bilal et al., 2018b
Shikimate	*E. coli*	Glycerol	Batch	5.33 g/L	24	Lee et al., 2017
Shikimate	*E. coli*	Glucose	Batch and fed-batch	1.73 g/L (Batch) 13.15g/L (Fed batch)	54	Gu et al., 2016
Shikimate	*E. coli*	Glycerol + glucose	Fed-batch	4.14 g/L (Batch) 27.41 g/L (Fed-batch)	48	Liu et al., 2016
Shikimate	*E. coli*	Glucose	Batch	3.12 g/L	–	Cui et al., 2014
Shikimate	*E. coli*	Glucose	Batch and fed-batch	1.12 g/L (Batch) 14.6 g/L (Fed-batch)	–	Chen et al., 2014
Shikimate	*E. coli*	Glycerol	Fed-batch	1.85 g/L	44	Chen et al., 2012
Shikimate	*Pichia stipites*	Glucose	Batch	3.11 g/L	120	Gao et al., 2017
Shikimate	*Corynebacterium glutamicum*	Glucose	Fed-batch	141 g/L	48	Kogure et al., 2016
MA	*E. coli*	Glucose, xylose	Fed-batch	4.7 g/L	72	Zhang et al., 2015
MA	*E. coli*	Glucose, glycerol	Batch	390 mg/L	32	Sun et al., 2013
MA	*E. coli*	Glucose, glycerol	Batch	1.5 g/L	48	Lin et al., 2014
MA	*E. coli*	Glucose	Batch	170 mg/L	72	Sengupta et al., 2015
MA	*Saccharomyces cerevisiae*	Glucose	Batch	1.56 mg/L	170	Weber et al., 2012
MA	*S. cerevisiae*	Glucose	Batch	141 mg/L	108	Curran et al., 2013
DHS	*E. coli*	Glucose	Batch	25.48 g/L	62	Yuan et al., 2014
DHS	*E. coli*	Glucose	Fed-batch	69g/L	–	Li et al., 1999

In this study, a phosphate-limited fed-batch fermentation process was successfully developed for enhanced production of DHS using a DHS-overproducing *E. coli* strain constructed by redesigning its biosynthetic pathway. The phosphate-limited fed-batch operation in the 7 L bioreactor level was observed to strictly regulate the specific growth rate (μ) and the glucose consumption rate and suppresses the production of undesirable byproducts by effectively controlling DO levels. Notably, under this culture conditions, DHS was biosynthesized in a non-growth-associated mode during the later stage of the fed-batch operations, efficiently utilizing higher portion of the supplied nutrients into the DHS biosynthesis rather than cellular growth. As a result, the Inha 103 strain showed significantly high level of DHS production amounting to 117 g/L (at a production yield (Yp/s) of 0.39 g/g glucose) in the 7-L fed-batch bioreactor culture, ~13-fold increase as compared to the parallel flask culture (9 g/L) performed under the identical culture conditions.

It was observed that sufficient amount of dissolved oxygen (DO) should be supplied in the cultivation of the metabolically engineered *E. coli* cells for enhanced production of DHS: Increase in oxygen mass transfer rate (kLa) through increment of agitation speed (rpm), and expansion of the diameter ratio of the impeller to the fermenter vessel up to 0.46 resulted in higher productivity of DHS. In addition, as described in this paper, by installing a turbine impeller just above the ring-type sparger for efficient break-down of the sparged air, and a marine impeller above the turbine impeller for proficient mixing of the dissolved oxygen, DO levels could be successfully maintained above 50% during the whole fermentation period, thereby leading to overcoming the undesirable oxygen-limited conditions due to relatively high cell density. Notably, with almost the same configuration of this fermenter system, higher production levels of MA (muconic acid) had been observed, maintaining DO levels above 40% during the entire fermentation time, leading to facilitated transfer of DO to the producing cells, and thereby improving MA production (Figure 4). In addition, by carefully controlling the composition and feeding rate of the supplied medium during the fed-batch operation, it was possible to overcome DO-limited conditions, simultaneously minimizing the production of the byproducts caused by high levels of residual glucose in the fermentation broth. Notably, under the phosphate-limited fed-batch fermentations, DHS was observed to be biosynthesized almost in a growth-associated mode, thus resulting in the remarkable enhancement in DHS productivity (i.e., ~6-fold increase as compared to the parallel batch bioreactor fermentation performed under the identical environments).

Such a remarkable production yield could be achieved by applying a combination of several metabolic engineering strategies for DHS production to *E. coli* strains. We demonstrated that engineering of key genes for DHS production using the host strain *E. coli* AB2834, in which the *aroE*-encoded shikimate dehydrogenase is inactivated, gradually increased the DHS production. We inactivated the *tyrR*-encoded tyrosine-dependent transcriptional regulator, which is a feedback repressor of *aroG*- and *aroF*-encoding DAHP synthase, for improving the transcription levels. Among the several approaches to improve PEP availability for promoting DHS production, we generated PTS-inactivated strain by disrupting *ptsG*, which is a PEP-dependent glucose transporter. Instead, we overexpressed *galP*-encoding D-galactose transporter, which is another glucose-uptake route. In addition, *pykA*-encoding pyruvate kinase 2 was removed and *ppsA*-encoded PEP synthase was overexpressed for reconversion of pyruvate to PEP. Furthermore, we overexpressed the genes on the biosynthetic pathway to complete the DHS-overproducing strain. Construction of an MA-producing *E. coli* cell factory was carried out by the introduction of a single operon containing *asbF*^*Eopt*^ *-aroY*^*Eopt*^ *-catA*^*Eopt*^ gene cluster, as well as applying a promoter engineering strategy through the transcriptome analysis. Although the current heterologous gene cluster converts MA to about 50% of DHS, strengthening and optimization of the DHS-PCA-CA-MA route in further studies could result in better performing strains with higher DHS-to-MA bioconversion efficiency.

In summary, we report the construction of *E. coli* strains capable of producing DHS at high concentrations from D-glucose. To accumulate high concentrations of DHS, *tyrR, ptsG*, and *pykA* gene were sequentially deleted from *E. coli* AB2834, in which the *aroE* gene was mutated to prevent the conversion of DHS to SA. Extra copies of *aroB, aroD, galP, ppsA, aroG*, and *aroF* genes involved in DHS biosynthesis were additionally inserted to maximize DHS accumulation. MA was also successfully produced by a heterologous expression pathway for DHS-to-MA bioconversion. A controlled fed-batch operation was performed with a statistically optimized production medium in a 7L bioreactor and the redesigned *E. coli* strain could convert DHS to MA efficiently, thereby producing about 64.5 g/L MA with almost no accumulation of metabolic intermediates such as PCA, CA, and DHS. This study demonstrates the potential value of *E. coli* host to produce high level of an intermediate metabolite of aromatic pathways and the rational cell factory design approach to possibly complement petrochemical-based chemical processes.

## Data Availability Statement

The datasets generated for this study are available on request to the corresponding author.

## Author Contributions

SK, SL, G-TC, and E-SK designed the research and provided the improvement of the manuscript. S-SC, S-YS, S-OP, H-NL, JS, JK and J-HP performed the experiments, as well as data collection and analysis. JS and JK performed RNA-seq analysis. S-SC and H-NL performed genetic engineering. S-YS and S-OP performed medium optimization and fermentation. S-SC and S-YS wrote the article.

### Conflict of Interest

S-YS, S-OP, H-NL, and SL were employed by the company STR Biotech Co., Ltd. The remaining authors declare that the research was conducted in the absence of any commercial or financial relationships that could be construed as a potential conflict of interest.

## References

[B1] Becker J. Wittmann C. (2015). Advanced biotechnology: metabolically engineered cells for the bio-based production of chemicals and fuels, materials, and health-care products. Angew. Chem. Int. Ed Engl. 54, 3328–3350. 10.1002/anie.201409033 25684732

[B2] Bilal M. Wang S. Iqbal H. M. N. Zhao Y. Hu H. Wang W.. (2018a). Metabolic engineering strategies for enhanced shikimate biosynthesis: current scenario and future developments. Appl. Microbiol. Biotechnol. 102, 7759–7773. 10.1007/s00253-018-9222-z 30014168

[B3] Bilal M. Yue S. Hu H. Wang W. Zhang X. (2018b). Systematically engineering *Escherichia coli* for enhanced shikimate biosynthesis co-utilizing glycerol and glucose. Biofuels Bioprod. Biorefin. 12, 348–361. 10.1002/bbb.1867

[B4] Bongaerts J. Krämer M. Müller U. Raeven L. Wubbolts M. (2001). Metabolic engineering for microbial production of aromatic amino acids and derived compounds. Metab. Eng. 3, 289–300. 10.1006/mben.2001.0196 11676565

[B5] Chandran S. S. Yi J. Draths K. M. von Daeniken R. Weber W. Frost J. W. (2003). Phosphoenolpyruvate availability and the biosynthesis of shikimic acid. Biotechnol. Prog. 19, 808–814. 10.1021/bp025769p 12790643

[B6] Chen K. Dou J. Tang S. Yang Y. Wang H. Fang H.. (2012). Deletion of the *aroK* gene is essential for high shikimic acid accumulation through the shikimate pathway in E. coli. Bioresour. Technol. 119, 141–147. 10.1016/j.biortech.2012.05.100 22728194

[B7] Chen X. Li M. Zhou L. Shen W. Algasan G. Fan Y.. (2014). Metabolic engineering of *Escherichia coli* for improving shikimate synthesis from glucose. Bioresour. Technol. 166, 64–71. 10.1016/j.biortech.2014.05.035 24905044

[B8] Chen Y. Nielsen J. (2013). Advances in metabolic pathway and strain engineering paving the way for sustainable production of chemical building blocks. Curr. Opin. Biotechnol. 24, 965–972. 10.1016/j.copbio.2013.03.008 23541505

[B9] Cheng L. Wang J. Xu Q. Xie X. Zhang Y. Zhao C. (2012). Effect of feeding strategy on L-tryptophan production by recombinant *Escherichia coli*. Ann. Microbiol. 62, 1625–1634. 10.1007/s13213-012-0419-6

[B10] Cui Y. Y. Chen L. Zhang Y. Y. Jian H. Liu J. Z. (2014). Production of shikimic acid from *Escherichia coli* through chemically inducible chromosomal evolution and cofactor metabolic engineering. Microb. Cell Fact. 13:21. 10.1186/1475-2859-13-21 24512078 PMC3923554

[B11] Curran K. A. Leavitt J. M. Karim A. S. Alper H. S. (2013). Metabolic engineering of muconic acid production in *Saccharomyces cerevisiae*. Metab. Eng. 15, 55–66. 10.1016/j.ymben.2012.10.003 23164574

[B12] Draths K. M. Frost J. W. (1994). Environmentally compatible synthesis of adipic acid from D-glucose. J. Am. Chem. Soc. 116, 399–400. 10.1021/ja00080a057

[B13] Gao M. Cao M. Suástegui M. Walker J. Rodriguez Quiroz N. Wu Y.. (2017). Innovating a nonconventional yeast platform for producing shikimate as the building block of high-value aromatics. ACS. Synth. Biol. 6, 29–38. 10.1021/acssynbio.6b00132 27600996

[B14] Gu P. Su T. Wang Q. Liang Q. Qi Q. (2016). Tunable switch mediated shikimate biosynthesis in an engineered non-auxotrophic *Escherichia coli*. Sci. Rep. 6:29745. 10.1038/srep29745 27406890 PMC4942831

[B15] Jan C. J. B. Cavallaro S. (2015). Transiting from adipic acid to bioadipic acid. 1, Petroleum-based processes. Ind. Eng. Chem. Res. 54, 1–46. 10.1021/ie5020734

[B16] Johansson L. Lindskog A. Silfversparre G. Cimander C. Nielsen K. F. Lidén G. (2005). Shikimic acid production by a modified strain of *E. coli* (W3110.shik1) under phosphate-limited and carbon-limited conditions. Biotechnol. Bioeng. 92, 541–552. 10.1002/bit.20546 16240440

[B17] Kogure T. Kubota T. Suda M. Hiraga K. Inui M. (2016). Metabolic engineering of *Corynebacterium glutamicum* for shikimate overproduction by growth-arrested cell reaction. Metab. Eng. 38, 204–216. 10.1016/j.ymben.2016.08.005 27553883

[B18] Kruyer N. S. Peralta-Yahya P. (2017). Metabolic engineering strategies to bio-adipic acid production. Curr. Opin. Biotechnol. 45,136–143. 10.1016/j.copbio.2017.03.006 28365404

[B19] Lee H. N. Shin W. S. Seo S. Y. Choi S. S. Song J. S. Kim J. Y.. (2018). *Corynebacterium* cell factory design and culture process optimization for muconic acid biosynthesis. Sci. Rep. 8:18041. 10.1038/s41598-018-36320-4 30575781 PMC6303301

[B20] Lee M. Y. Hung W. P. Tsai S. H. (2017). Improvement of shikimic acid production in *Escherichia coli* with growth phase-dependent regulation in the biosynthetic pathway from glycerol. World J. Microbiol. Biotechnol. 33:25. 10.1007/s11274-016-2192-3 28044275

[B21] Li K. Mikola M. R. Draths K. M. Worden R. M. Frost J. W. (1999). Fed-batch fermentor synthesis of 3-dehydroshikimic acid using recombinant *Escherichia coli*. Biotechnol. Bioeng. 64, 61–733. 10.1002/(SICI)1097-0290(19990705)64:1<61::AID-BIT7>3.0.CO;2-G 10397840

[B22] Li L. Tu R. Song G. Cheng J. Chen W. Li L.. (2019). Development of a synthetic 3-dehydroshikimate biosensor in *Escherichia coli* for metabolite monitoring and genetic screening. ACS Synth. Biol. 8, 297–306. 10.1021/acssynbio.8b00317 30609888

[B23] Lin Y. Sun X. Yuan Q. Yan Y. (2014). Extending shikimate pathway for the production of muconic acid and its precursor salicylic acid in Escherichia coli. Metab. Eng. 23, 62–69. 10.1016/j.ymben.2014.02.009 24583236

[B24] Liu X. Lin J. Hu H. Zhou B. Zhu B. (2016). Site-specific integration and constitutive expression of key genes into *Escherichia coli* chromosome increases shikimic acid yields. Enzyme Microb. Technol. 82, 96–104. 10.1016/j.enzmictec.2015.08.018 26672454

[B25] Martínez J. A. Bolívar F. Escalante A. (2015). Shikimic acid production in *Escherichia coli*: from classical metabolic engineering strategies to omics applied to improve its production. Front. Bioeng. Biotechnol. 3:145. 10.3389/fbioe.2015.00145 26442259 PMC4585142

[B26] Meza E. Becker J. Bolivar F. Gosset G. Wittmann C. (2012). Consequences of phosphoenolpyruvate sugar phosphotranferase system and pyruvate kinase isozymes inactivation in central carbon metabolism flux distribution in *Escherichia coli*. Microb. Cell Fact. 11:127. 10.1186/1475-2859-11-127 22973998 PMC3521201

[B27] Nishikura-Imamura S. Matsutani M. Insomphun C. Vangnai A. Toyama H. Yakushi T.. (2014). Overexpression of a type II 3-dehydroquinate dehydratase enhances the biotransformation of quinate to 3-dehydroshikimate in *Gluconobacter oxydans*. Appl. Microbiol. Biotechnol. 98, 2955–2963. 10.1007/s00253-013-5439-z 24352733

[B28] Niu W. Draths K. M. Frost J. W. (2002). Benzene-free synthesis of adipic acid. Biotechnol. Prog. 18, 201–211. 10.1021/bp010179x 11934286

[B29] Noda S. Kondo A. (2017). Recent advances in microbial production of aromatic chemicals and derivatives. Trends Biotechnol. 35, 785–796. 10.1016/j.tibtech.2017.05.006 28645530

[B30] Patnaik R. Spitzer R. G. Liao J. C. (1995). Pathway engineering for production of aromatics in *Escherichia coli*: confirmation of stoichiometric analysis by independent modulation of *AroG, TktA*, and *Pps* activities. Biotechnol. Bioeng. 46, 361–370. 10.1002/bit.260460409 18623323

[B31] Pittard J. Yang J. (2008). Biosynthesis of the aromatic amino acids. EcoSal Plus 3, 1–39. 10.1128/ecosalplus.3.6.1.8 26443741

[B32] Richman J. E. Chang Y. Kambourakis S. Draths K. M. Almy E. Snell K. D. (1996). Reaction of 3-dehydroshikimic acid with molecular oxygen and hydrogen peroxide: products, mechanism, and associated antioxidant activity. J. Am. Chem. Soc. 118, 11587–11591. 10.1021/ja952317i

[B33] Rodriguez A. Martínez J. A. Báez-Viveros J. L. Flores N. Hernández-Chávez G. Ramírez O. T.. (2013). Constitutive expression of selected genes from the pentose phosphate and aromatic pathways increases the shikimic acid yield in high-glucose batch cultures of an *Escherichia coli* strain lacking PTS and *pykF*. Microb. Cell Fact. 12:8. 10.1186/1475-2859-12-86 24079972 PMC3852013

[B34] Rodriguez A. Martínez J. A. Flores N. Escalante A. Gosset G. Bolivar F. (2014). Engineering *Escherichia coli* to overproduce aromatic amino acids and derived compounds. Microb. Cell Fact. 13:126. 10.1186/s12934-014-0126-z 25200799 PMC4174253

[B35] Sengupta S. Jonnalagadda S. Goonewardena L. Juturu V. (2015). Metabolic engineering of a novel muconic acid biosynthesis pathway via 4-hydroxybenzoic acid in *Escherichia coli*. Appl. Environ. Microbiol. 81, 8037–8043. 10.1128/AEM.01386-15 26362984 PMC4651072

[B36] Sun X. Lin Y. Huang Q. Yuan Q. Yan Y. (2013). A novel muconic acid biosynthesis approach by shunting tryptophan biosynthesis via anthranilate. Appl. Environ. Microbiol. 79, 4024–4030. 10.1128/AEM.00859-13 23603682 PMC3697559

[B37] Tzin V. Galili G. (2010). The biosynthetic pathways for shikimate and aromatic amino acids in *Arabidopsis thaliana*. Arabidopsis Book 8:e0132. 10.1199/tab.0132 22303258 PMC3244902

[B38] Wang J. Cheng L. Wang J. Liu Q. Shen T. Chen N. (2013). Genetic engineering of *Escherichia coli* to enhance production of L-tryptophan. Appl. Microbiol. Biotechnol. 97, 7587–7596. 10.1007/s00253-013-5026-3 23775271

[B39] Weber C. Brückner C. Weinreb S. Lehr C. Essl C. Boles E. (2012). Biosynthesis of *cis,cis*-muconic acid and its aromatic precursors, catechol and protocatechuic acid, from renewable feedstocks by *Saccharomyces cerevisiae*. Appl. Environ. Microbiol. 78, 8421–8430. 10.1128/AEM.01983-12 23001678 PMC3497357

[B40] Yi J. Li K. Draths K. M. Frost J. W. (2002). Modulation of phosphoenolpyruvate synthase expression increases shikimate pathway product yields in E. coli. Biotechnol. Prog. 18, 1141–1148. 10.1021/bp020101w 12467444

[B41] Yuan F. Chen W. Jia S. Wang Q. (2014). Improving 3-dehydroshikimate production by metabolically engineered *Escherichia coli*. Sheng Wu Gong Cheng Xue Bao 30, 1549–1560. 10.13345/j.cjb.140019 25726580

[B42] Zhang H. Pereira B. Li Z. Stephanopoulos G. (2015). Engineering *Escherichia coli* coculture systems for the production of biochemical products. Proc. Natl. Acad. Sci. U.S.A. 112, 8266–8271. 10.1073/pnas.1506781112 26111796 PMC4500268

