# Supplementary material for: Cell Factory Design and Culture Process Optimization for Dehydroshikimate Biosynthesis in Escherichia coli
Source: Front Bioeng Biotechnol. 2019 Oct 9;7:241. doi: 10.3389/fbioe.2019.00241 (PMC6795058; doi:10.3389/fbioe.2019.00241)

Cell Factory Design and Culture Process Optimization for Dehydroshikimate Biosynthesis in *Escherichia coli*

Si-Sun Choi[1&], Seung-Yeul Seo[2,4&], Sun-Ok Park[2], Han-Na Lee[1,2], Ji-soo Song[1], Ji-yeon Kim[1], Ji-Hoon Park[1], Sangyong Kim[3,5], Sang Joung Lee[2], Gie-Taek Chun[4,*], and Eung-Soo Kim[1,*]

[1]Department of Biological Engineering, Inha University, Incheon 22212, Republic of Korea

[2]STR Biotech Co., Ltd., Bioplaza 4-3, 56, Soyanggang-ro, Chuncheon-si, Gangwon-do 24232, Republic of Korea

[3]Green Chemistry and Materials Group, Korea Institute of Industrial Technology, Cheonan si, Chungcheongnam-do, 31056, Republic of Korea

[4]Department of Molecular Bio-science, Kangwon National University, Chuncheon-si, Gangwon-do 24341, Republic of Korea

[5]Green Process and System Engineering Major, Korea University of Science and Technology (UST), Daejeon, 34141, Republic of Korea.

[&]Equal contribution

[*]Corresponding authors: Eung-Soo Kim & Gie-Taek Chun

Supplementary

Table 1. PCR primers of *E. coli* study for construction of plasmids and genome confirm

Primer	Forward(F) / Reverse(R)	Target
R1	F 5'- CAGGTGATGGATGTCGACAAACCACTACCG – 3'	*tyrR*
R2	R 5'- TCGACAGAGAGCAAAGCTTCAGGCAACGCC – 3'	
R3	F 5'- ACTGACACAACTCGAGGGTTATGAGCTGCG – 3'	
R4	R 5'- GCATCGCAACGCCTGGATCCGCCAATAGCT – 3'	
G1	F 5'- TTACATATGCGGGATCCGGTAGGCGAACGT – 3'	*ptsG*
G2	R 5'- CATGGTTTTAACCATCTAGACATAGGCAACAACTCGAGCCAGCGCGGATA – 3'	
G3	F 5'- TCCACGCGATTCTAGAAGGCCTGGCATTCCCAAGCTTTATTCTTCTGGGG – 3'	
G4	R 5'- GTCGACCTACGCCAGCTATA – 3'	
A1	F 5'- CAACCGCGCCGTCGACTTGCTC – 3'	*pykA*
A2	R 5'- CAAACGGCTTCTAGACGTTCAAGCTTGGCAACAA– 3'	
A3	F 5'- CGTTTCTAGACACCGTCTCGAGGTTCAGTTCGAC – 3'	
A4	R 5'- CCGCCAAGGATCCGTGATCCCATTCT – 3'	
I1	F 5'- ATTCGGATCCTGCTCATCCATGACCTGACC -3'	*lacI*
I2	R 5'- GAACTCTAGATAATGCAGCTGGCACGACAG -3'	
I3	F 5'- ATGGTCTAGAGAATGTAATTCAGCTCCGCC -3'	
I4	R 5'- ATGCGTCGACTTGTGCGCTCAGTATAGGAAG -3'	
Plac1	F 5'- GAGGGGATCCAGATCGCGGCCGCGCAATTAATGTGAGTTAGCTC -3'	pUC18
Plac2	R 5'- TCTAGAAATAATTTTAATTGTTATCCGCTCACAAT -3'	
RBS1	F 5'- TCTAGAAATAATTTTGTTTA -3'	pET-21b(+)
RBS2	R 5'- GGCGAATTCCAAGCTCATATGTATATCTCCTTCTT -3'	
B1	F 5'- TAAGAAGGAGATATACATATGGAGAGGATTGTCGTTACT -3'	*E. coli* K12 Genomic DNA
B2	R 5'- CAGTTACGGTTTTCATTACGCTGATTGACAATCGG -3'	
D1	F 5'- ATGAAAACCGTAACTGTAAAA -3'	
D2	R 5'- GGCGGGTGTCGGGGCAAGCTTTTATGCCTGGTGTAAAATAGTT -3'	
A3	F 5'- CTCCCGGCCGCCATGCACATAACCCCGGCGACTAA -3'	
A4	R 5'- CCCGCGGCCGCCATGCACAAAAGGATTGTTCGATG -3'	
P1	F 5'- AGGAGATATACTCGAATGCCTGACGCTAAAAAACA -3'	
P2	R 5'- GAATTCGATTCTCGAGGAGATTAATCGTGAGCGCC -3'	
G3	F 5'- AGGAGATATACTCGAATGAATTATCAGAACGACGA -3'	
G4	R 5'- GAATTCGATTCTCGATTACCCGCGACGCGCTTTTA -3'	
F1	F 5'- CTCCCGGCCGCCATGATAAACCTCTTAAGCCACGC -3'	
F2	R 5'- CCCGCGGCCGCCATGTACGTCATCCTCGCTGAGGA -3'	
BD1	F 5'- AATTACATTCTCTAGCGCAATTAATGTGAGTTAGC -3'	T-BD
BD2	R 5'- TCATGGTCATTCTAGTTATGCCTGGTGTAAAATAG -3'	
BD3	R 5'- CATATGGGACCTAGGTTATGCCTGGTGTAAAATAG -3'	
PA1	F 5'- CCTAGGTCCCATATGGTCGACCTGCA -3'	T-PA
PA2	R 5'- TCATGGTCATTCTAGTTATTTCTTCAGTTCAGCCA -3'	
GF1	F 5'- CCAGGCATAACCTAGTCCCATATGGTCGACCTGCA -3'	T-GF
GF2	R 5'- CCATATGGGACCTAGAAACCTCTTAAGCCACGCGA -3'	

Table 2. 25ml lab-scale culture. Pf, maximum DHS production (g IA/L); Xf, maximum dry cell weight (g DCW/L); Sf, final residual glucose concentration (g glucose/L); Yp/s, DHS production yield based on glucose (g DHS/g glucose); Qp, average volumetric DHS production rate (g DHS/L/h).

**Strain**	**Pf**	**Sf**	**Yp/s**	**Qp**
AB2834	3.54	0	0.708	0.037
Inha 24	4.92	0	0.984	0.051
Inha 29	5.82	0	1.164	0.061
Inha 52	7.72	0	1.544	0.080
Inha 99	8.06	0	1.612	0.084
Inha 95	9.20	0	1.84	0.096
Inha 103	9.28	0	1.856	0.097

Figure 1. Transcriptome analysis for promoter selection and pathway re-design. The red asterisk represents the selected promoter in this study (A) Cell growth of *E. coli* AB2834 and Inha52 (B) RNA expression analysis from early stage of exponential phase (AB2834, 4h; Inha52, 7h) (C) RNA expression analysis from the early stage of stationary phase (AB2834, 9h; Inha52, 12h).

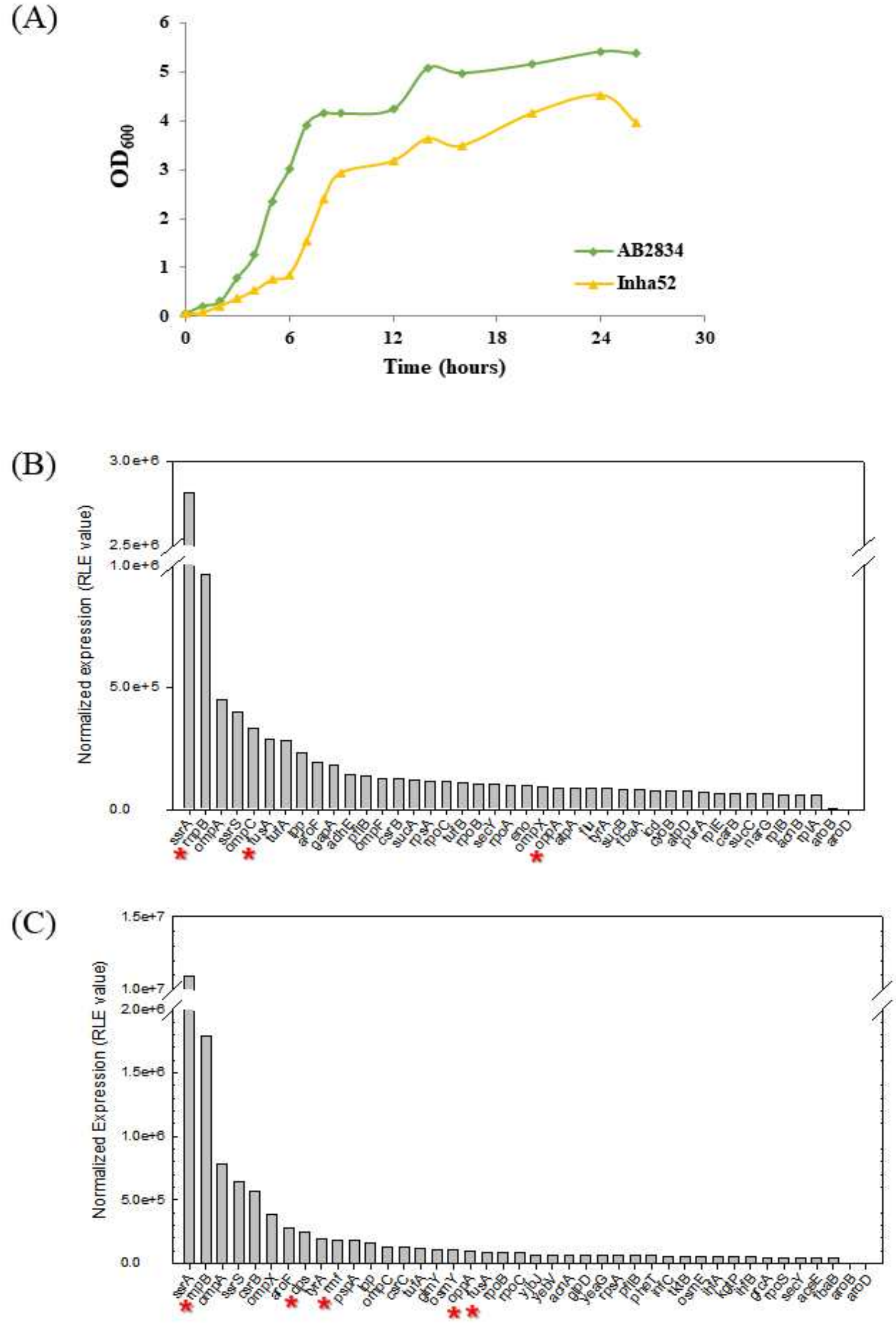

Figure 2. **(A)** Schematic representation of the operons, including codon-optimized MA biosynthetic genes (*asbF*^{Eopt}, *aroY*^{Eopt}, *catA*^{Eopt}) constructed in this study. **(B)** Production of metabolites by *E. coli* strains harboring gene operons (presented in panel A). *Red,* DHS; *Green,* PCA; *Grey,* MA.

(A)

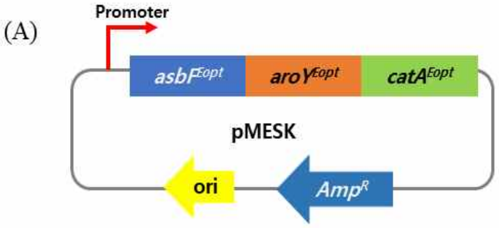

Plasmid	Promoter	Promoter description
pMESK1	*plac*	Lac promoter in PUC18
pMESK4	P*oppA*	oligopeptide ABC transporter periplasmic binding protein
pMESK5	P*dps*	Fe-binding and storage protein; stress-inducible DNA-binding protein
pMESK6	P*rmf*	ribosome modulation factor
pMESK7	P*fusA*	protein chain elongation factor EF-G, GTP-binding
pMESK8	P*rnpB*	RNA component of RNase P

(B)

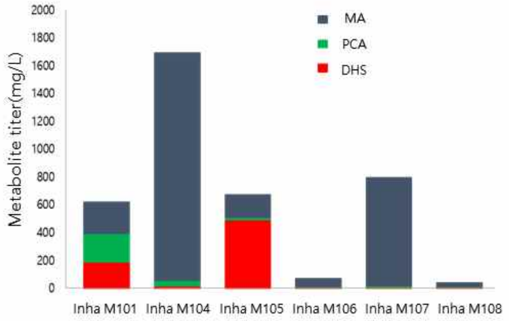

Figure 3. Time course profiles of cell growth (A) and DHS production(B) by serial engineered strains in the 7-L fermenter.

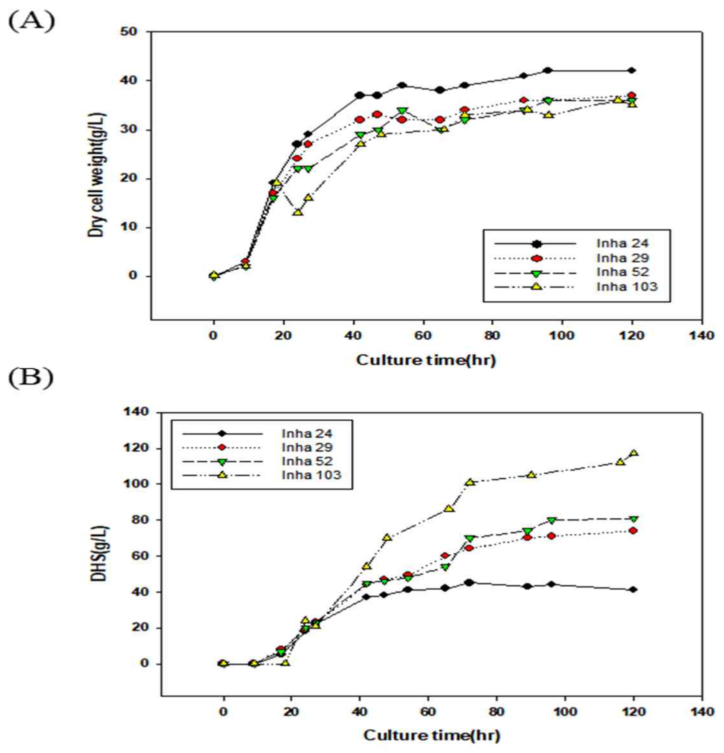

Figure 4. Gene expression level based on the results of RNA-seq by time course (time 1; 7hr, time 2; 12hr)

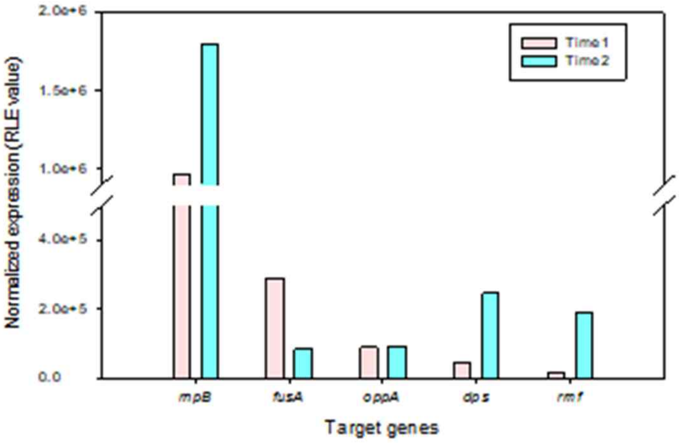

Supplement: Supplementary file 1 [file Data_Sheet_1.PDF]
